# A novel intelligent physiotherapy robot based on dynamic acupoint recognition method

**DOI:** 10.3389/fnbot.2025.1696824

**Published:** 2025-11-24

**Authors:** Yuhan Zhang, Shiyang Sun, Donghui Zhao, Junyou Yang, Shuoyu Wang

**Affiliations:** 1 School of Electrical Engineering, Shenyang University of Technology, Shenyang, China; 2 Department of Intelligent Mechanical System Engineering, Kochi University of Technology, Kochi, Japan

**Keywords:** physiotherapy robot, acupoint recognition, RTMDet network, RTMPose network, physiotherapy task

## Abstract

**Background:**

Physiotherapy robots offer a feasible and promising solution for achieving safe and efficient treatment. Among these, acupoint recognition is the core component that ensures the precision of physiotherapy robots. Although the research on the acupoint recognition such as hand and ear has been extensive, the accurate location of acupoints on the back of the human body still faces great challenges due to the lack of significant external features.

**Methods:**

This paper designs a two-stage acupoint recognition method, which is achieved through the cooperation of two detection networks. First, a lightweight RTMDet network is used to extract the effective back range from the image, and then the acupoint coordinates are inferred from the extracted back range, reducing the inference consumption caused by invalid information. In addition, the RTMPose network based on the SimCC framework converts the acupoint coordinate regression problem into a classification problem of sub-pixel block subregions on the X and Y axes by performing sub-pixel-level segmentation of images, significantly improving detection speed and accuracy. Meanwhile, the multi-layer feature fusion of CSPNeXt enhances feature extraction capabilities. Then, we designed a physiotherapy interaction interface. Through the three-dimensional coordinates of the acupoints, we independently planned the physiotherapy task path of the physiotherapy robot.

**Results:**

We conducted performance tests on the acupoint recognition system and physiotherapy task planning in the physiotherapy robot system. The experiments have proven our effectiveness, achieving a recall of 90.17% on human datasets, with a detection error of around 5.78 mm. At the same time, it can accurately identify different back postures and achieve an inference speed of 30 FPS on a 4070Ti GPU. Finally, we conducted continuous physiotherapy tasks on multiple acupoints for the user.

**Conclusion:**

The experimental results demonstrate the significant advantages and broad application potential of this method in improving the accuracy and reliability of autonomous acupoint recognition by physiotherapy robots.

## 1 Introduction

With the continuous deepening of population aging and the improvement of public health awareness, traditional Chinese medicine, as a natural therapy for regulating body functions, is gradually attracting more and more attention. Chinese medicine therapy stimulates specific acupoints to trigger local and systemic sensory mechanisms and biological responses in the body, and has significant therapeutic effects in relieving various types of pain, promoting blood circulation, improving physical comfort, and psychological relaxation (Kerautret et al., 2024; Li et al., 2025). However, traditional physical therapy services are expensive, vary in quality, and suffer from a shortage of professional therapists, making it difficult to meet growing health demands. As an innovative product integrating artificial intelligence, robotics technology and traditional Chinese medicine theory, physiotherapy robots offer a new solution and development direction to address this issue (Pang et al., 2019; Zhao et al., 2023). Several countries and regions have successively introduced relevant support policies, providing strong financial support and resource guarantees for research in this field, effectively promoting the application of physiotherapy robots in clinical rehabilitation, home nursing, and other scenarios (Zhao and Guo, 2023). Currently, there are a variety of physiotherapy robot products on the market that offer a range of functions (Yang et al., 2024), including massage, moxibustion, and tuina massage, among other traditional Chinese medicine physiotherapy methods. The level of intelligence is constantly improving, gradually promoting the development of intelligent physiotherapy toward personalization and precision.

Therapeutic acupoints are mainly distributed along the human meridian system and are a core component of traditional Chinese medicine theory. Physiotherapy robots rely on the precise localization of human acupoints to effectively exert their therapeutic effects (Yu et al., 2024). However, there are still many challenges in achieving autonomous acupoint recognition, especially in locating acupoints on the back. Compared to areas such as the face or limbs, the back lacks obvious structural landmarks, has relatively smooth skin texture, and varies greatly between individuals (Yang et al., 2025a). Therefore, how to improve the accuracy of physiotherapy robots in acupoint recognition on the back remains one of the challenges that need to be overcome in its development. In this paper, we propose an acupoint recognition method for autonomous physical therapy robot systems that combines high accuracy and dynamic performance to automatically identify patients’ acupoints. We systematically modeled the location of acupoints on the back as a key point recognition task and trained the two-stage neural network through our own established dataset to predict the relevant acupoints. And this acupoint recognition network was applied to the physiotherapy robot system built by oneself, enabling the physiotherapy robot to perform physiotherapy tasks automatically. Our main contributions are as follows:

- An indirect data annotation method is proposed. Firstly, manual annotation is carried out on the human body, then software annotation is conducted using annotation software, and finally the manual marks on the images are removed through algorithms to systematically improve the consistency of annotation and the quality of training data.
- Based on the two-stage network architecture, a key point prediction network for back physiotherapy acupoints was designed, that is, the combination of target range detection and key point detection was used for physiotherapy acupoint recognition, and it was applied to the self-built physiotherapy robot system, which can significantly improve the accuracy and objectivity of localization.

## 2 Related work

### 2.1 Current research status of physiotherapy robots

Many universities and enterprises at home and abroad have invested in the research and development of traditional Chinese medicine physiotherapy robots. This field is gradually moving toward a composite direction that integrates visual perception, force control feedback, and multi-probe coordination (Xing et al., 2021; Liu et al., 2024). Wang et al. (2018) designed a compact, space-saving portable back massage robot that optimizes electromagnetic force distribution through the establishment of a 3D electromagnetic simulation model, ensuring that the massage robot can cover the entire back area and improve massage effectiveness. However, it does not have acupoint recognition capabilities and cannot perform precise physical therapy. However, physiotherapy robots still have insufficient adaptation to individual differences in acupoint recognition and certain limitations in robustness in complex application scenarios. Dong et al. (2022) designed a compliant parallel robotic massage arm that combines serial elastic actuators (SEA) to achieve unified force-position control without relying on a complete dynamic model. Sayapin (2017) developed an intelligent autonomous mobile massage robot. The device uses a three-degree-of-freedom motion system and a triangular topology design. It consists of three vacuum massage cups and linear drivers connected to the vacuum massage cups. It uses a sliding cupping massage method to slide and stretch muscle tissue to achieve a massage effect. Due to structural design limitations, the robot may not be able to adapt to the complex curves of the human body and does not have an acupoint positioning function. Choi et al. (2021) developed an abdominal massaging device named Bamk-001. This device is equipped with five thermoelectric modules and can stably provide a constant-temperature heat compress at 40 °C, bringing continuous warm therapeutic effects to the abdomen. Meanwhile, with the coordinated operation of the five airbags, rhythmic pressure is applied to the patient’s abdomen through a clockwise circulation of inflation, thereby simulating the technique of artificial massage. However, this device is unable to precisely locate human acupoints. Its massage effect is limited to the entire abdominal area and lacks targeted acupoint stimulation functions. Therefore, it has limitations in application scenarios such as traditional Chinese medicine meridian therapy that require precise positioning. Zhu et al. (2023) designed a peristaltic wearable massage robot for the treatment of diseases related to lymph and blood circulation. This robot uses a fluid fabric muscle plate as the driver and is driven by a hydraulic transmission device, which enables the robot to provide dynamic compressive force to meet the massage requirements even at higher frequencies. This robot mainly relies on a fixed compressed wave pattern to improve lymphatic or blood circulation and is unable to precisely identify the locations of human acupoints. Autonomous acupoint recognition by physical therapy robots is the key foundation for realizing personalized physical therapy plans. Accurate acupoint recognition not only directly affects the accuracy and effectiveness of physical therapy, but also provides a reliable basis for technique selection, force control, and treatment path planning. Especially for the back area, there are many acupuncture points distributed over a wide area, and their precise location is particularly critical for effective therapeutic intervention.

### 2.2 Research status of acupoint recognition method

With the rapid advancement of computer vision, deep learning and sensor technology, researchers have attempted to apply methods such as image recognition, 3D reconstruction, infrared thermal imaging and neural networks to acupoint recognition to enhance its accuracy and degree of automation (Liu et al., 2023; Huang et al., 2025). Mamieva et al. (2023) proposed using a binocular telescope structure as the basis for a keypoint detection network to improve model construction effectiveness. This framework demonstrated high average precision (AP) values under multi-scale inference strategies and achieved excellent facial detection results. However, the model’s ability to process blurry images in low-light environments still needs improvement, and it relies heavily on high-performance external devices. Malekroodi et al. (2024) applied a real-time landmark detection system to identify anatomical landmarks in images, convert their coordinates into spatial coordinates corresponding to acupoints, and locate 38 specific acupoints on the face and hands. He also used a convolutional neural network (CNN) specifically optimized for pose estimation and trained it with restricted medical imaging data, to detect five key acupoints on the arms and hands. Zheng (2022) developed a mobile augmented reality (AR) system based on a facial landmark recognition network, combining deep learning models with traditional Chinese medicine bone measurement methods to achieve real-time identification and localization of facial acupoints. However, in practical applications, this method has certain limitations when dealing with complex situations such as facial rotation, and it relies on complete facial image input. Yuan et al. (2024) improved the YOLOv8-pose key point detection algorithm for facial acupoints. By integrating ECA attention to enhance acupoint feature extraction and replacing the original neck module with a more lightweight Slim-neck module, they provided significant reference value for facial acupoint localization and detection. However, the self-built dataset in the paper fails to fully cover all kinds of extreme scenarios, which may limit the generalization ability of the model when facing the diverse application scenarios in the real world. Wang et al. (2023) constructed a cascaded network model using HRNet as the backbone and introduced a dual attention mechanism combining SE and CA, effectively enhancing the model’s ability to perceive local key features. Seo et al. (2024) focused on the problem of accurately locating acupoints in 2D hand images, comparing the performance of two deep learning architectures, HRNet and ResNet, to explore methods for improving the accuracy of hand acupoint detection. Yang et al. (2025b) proposed a hybrid model RT-DEMT, which combines the efficient state space model Mamba with a Transformer module based on the DETR architecture, improving the accuracy and speed of back acupoint localization. This model primarily relies on visual images as input and has not yet integrated multimodal information such as infrared, depth maps, or human body structure point clouds. Therefore, its recognition robustness in complex environments still has certain limitations. Zhang et al. (2024) proposed a method for detecting back acupoints based on an improved OpenPose, which enhanced the reasoning speed. However, the number of evaluated acupoints is relatively small, and the robustness verification in complex scenarios still needs to be improved. In conclusion, acupoint recognition on the back is of great significance in application scenarios such as moxibustion and physical therapy. Due to the diversity of postural changes on the human back, the design and proposal of an acupoint recognition algorithm that can adapt to postural changes and lighting interference is of great theoretical and practical significance for improving the therapeutic effects and application value of physical therapy robots.

### 2.3 Physiotherapy robot

**Figure 1:**
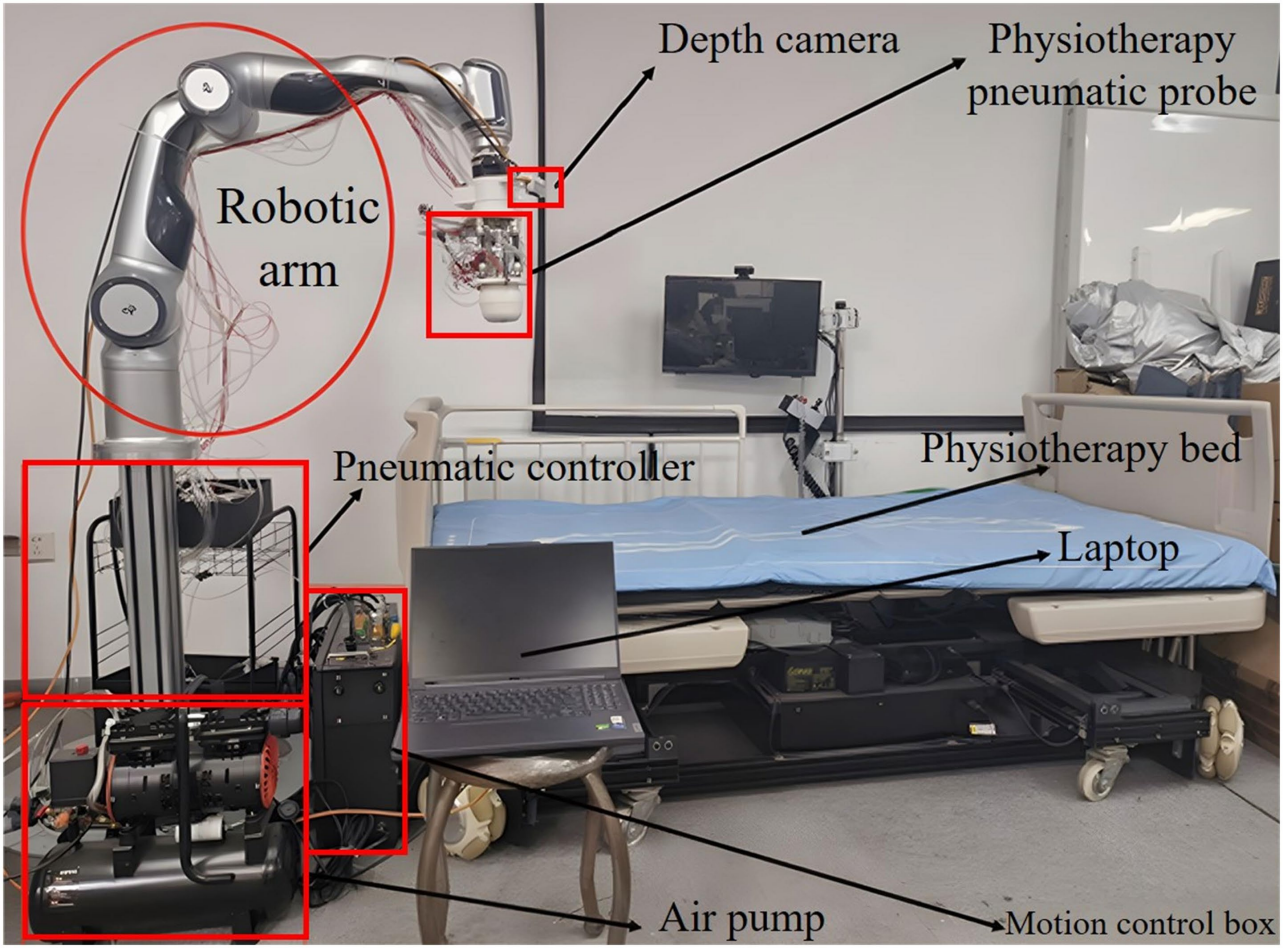
Structure diagram of the physiotherapy robot platform.

This paper establishes its own physical therapy robot hardware platform, as shown in Figure 1, which mainly consists of a physical therapy robotic arm, a robotic arm control box, a depth camera, a rigid-flexible coupling massage head and air pump, a computer, and a physiotherapy bed.

Among them, the robotic arm is a 7-degree-of-freedom series robotic arm, mainly serving as part of the physiotherapy execution mechanism to complete tasks such as acupoint recognition and reproducing manual techniques. At the end, it is equipped with a pneumatic rigid-flexible coupling probe, which has various forms such as palm and finger. The air pump is a high-pressure air pump equipped with a PWM control system, which can provide high-pressure gas of different frequencies and pressures, and switch and control the force through pneumatic means. The D435i depth camera is selected to obtain the real-time color RGB images of the physiotherapy bed and the corresponding depth image data, and feed them back to the computer. The computer is responsible for the operation of the human-computer interaction system, the acquisition of camera image data, as well as the acupoint recognition, the generation of control instructions for the mechanical arm, and the planning of physiotherapy tasks and other major calculations. The physiotherapy bed can undergo various posture changes and height adjustments.

## 3 Method

In this section, the proposed network structure and localization framework are systematically described for the problem of acupoint recognition on the back. In Section 3.1, the acquisition process of the experimental dataset and its preprocessing strategy are introduced in detail. In Section 3.2, the constructed acupoint recognition network framework is described in detail. In Section 3.3, evaluation metrics for performance verification are proposed and explained, providing standards for subsequent experimental analysis. Finally, in Section 3.4, the coordinate transformation and trajectory planning of robots when performing physiotherapy tasks are explained.

### 3.1 Data set and processing

Since deep learning is a data-driven predictive method, the quality of the dataset has a significant impact on model performance. In the current process of constructing acupoint datasets, software annotation directly on images or automatic annotation using mathematical models is commonly used. Due to factors such as projection distortion and camera nonlinear distortion, visual errors are inevitable during the annotation process, and some areas cannot be accurately annotated due to a lack of sufficient positioning information. For this purpose, this study proposes an indirect data annotation method. First, frontline Chinese medicine experts are invited to perform manual annotation on the human body. Then, annotation software is used for software annotation. Finally, an algorithm is used to remove the manual markings from the images. This study recruited 100 people as volunteers. Before the experiment began, they were all informed in detail of the research purpose, experimental procedures and related precautions, and voluntarily signed the informed consent form on the basis of full understanding. The participants’ body types were evaluated based on the body mass index (BMI). Before the experiment, their height and weight were obtained through questionnaires and calculated according to BMI. Among these volunteers, 2 had a BMI less than 18.5, 80 had a BMI between 18.5 and 24, 15 had a BMI between 24 and 28, and 3 had a BMI more than 28. Our sample covered all BMI stratifications, among which normal body types accounted for the majority.

**Figure 2:**
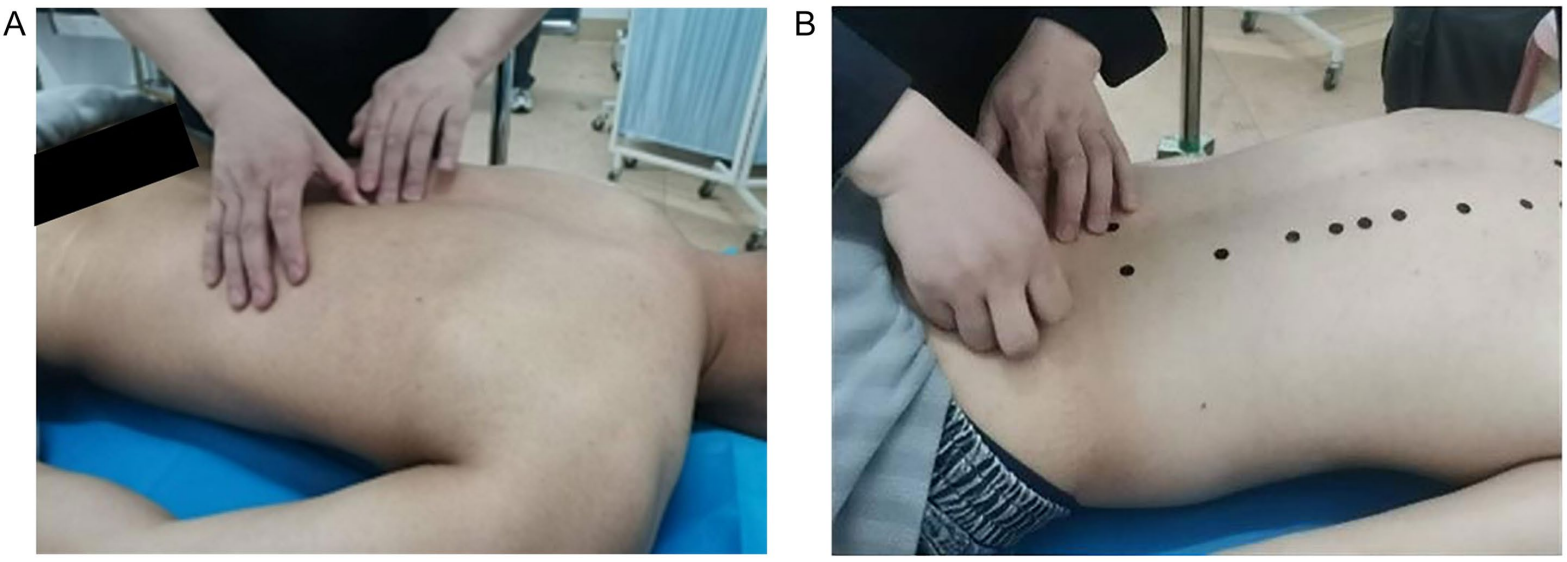
The process of experts locating acupoints and applying labels. **(A)** The process of locating the acupoint. **(B)** Label the acupoints.

**Figure 3:**
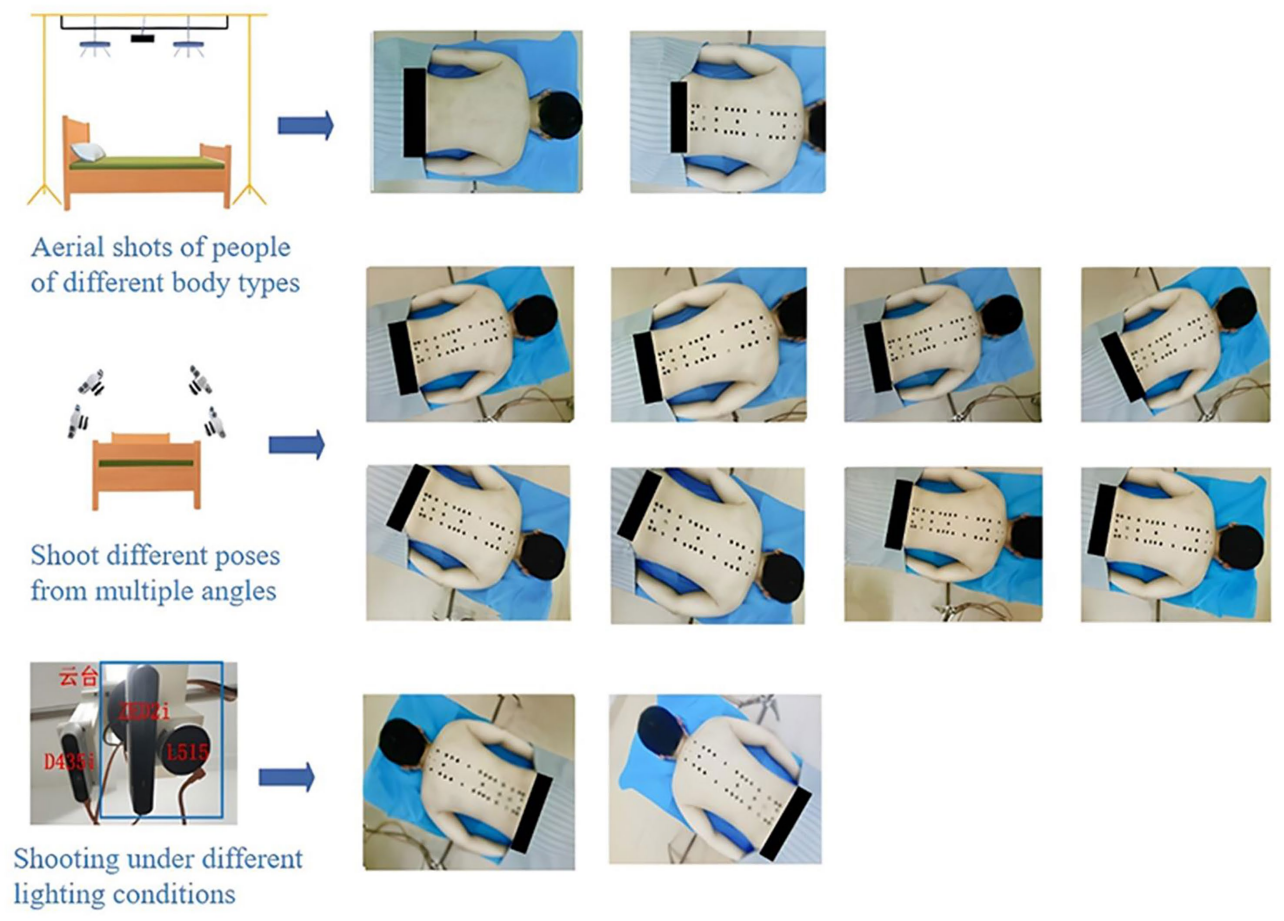
Multi-angle acquisition process of back acupoint images.

**Figure 4:**
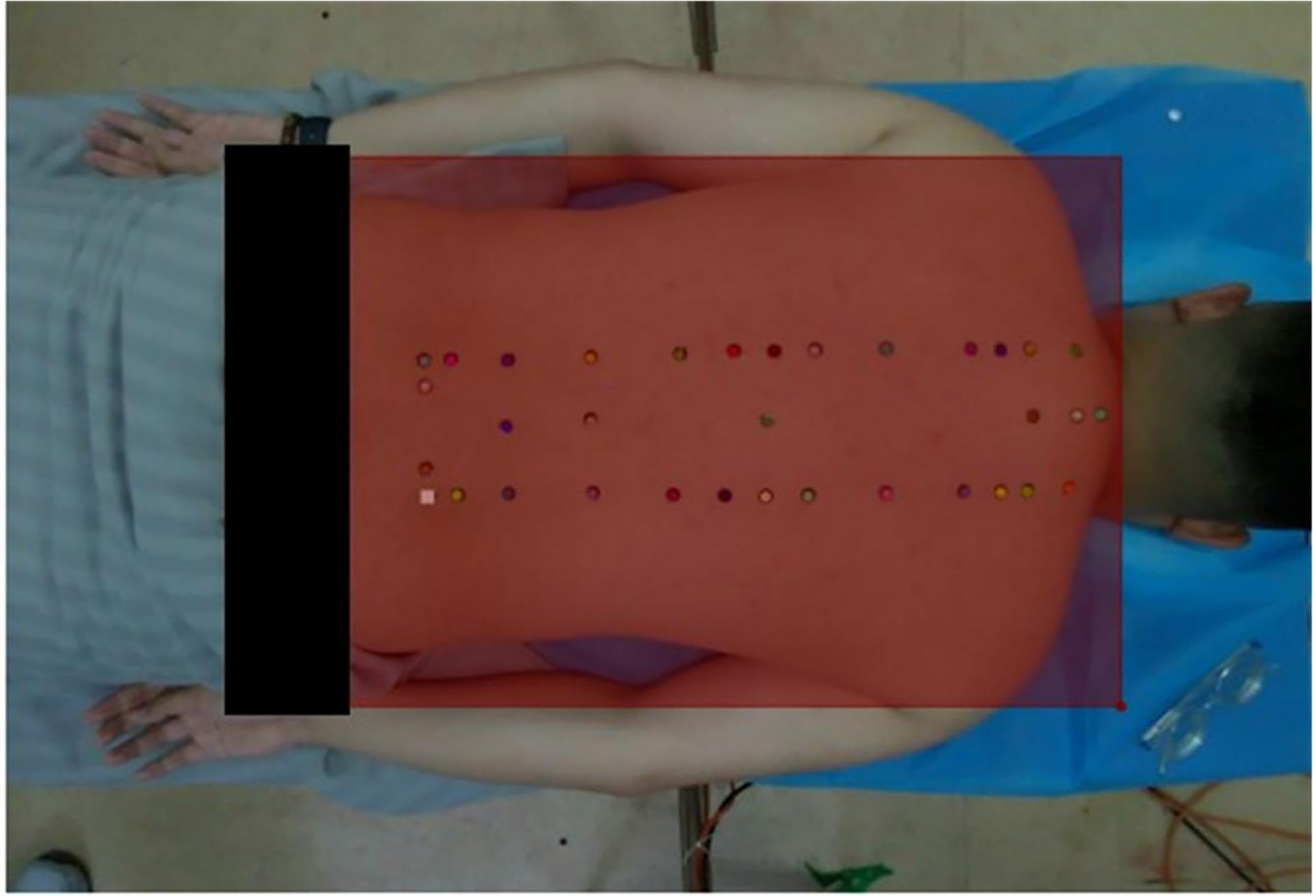
Annotation example.

Data collection was carried out in a well-lit laboratory. Three different types of depth cameras were used to obtain multi-resolution images, and the back postures of the volunteers were captured from multiple angles. The specific steps for data collection are as follows:

- Volunteers lay prone on the bed, removed their back clothing, and professional Chinese medicine experts identify their acupoints. For female subjects, the experiment was independently conducted by female professionals, and the subjects participated wearing underwear. After locating the acupoints, the experts mark the corresponding locations with round adhesive labels. As shown in Figure 2.
- After completing the acupoint marking, the camera gimbal was controlled by a self-written data collection program to capture images of the volunteer’s back from different angles, including horizontal, multi-angle horizontal rotation, and multi-angle tilt. After the program finished collecting data, a mobile phone was used to supplement the images from multiple random angles to enhance data diversity. As shown in Figure 3.
- To avoid the impact of factors such as angle, lighting changes, or annotation errors during data collection, the collected images must be manually screened. After screening, the Labelme annotation tool is used to precisely annotate the center points of circular labels in the images and extract the pixel coordinate information of the acupoints. As shown in Figure 4, it is an example of annotation.

**Figure 5:**
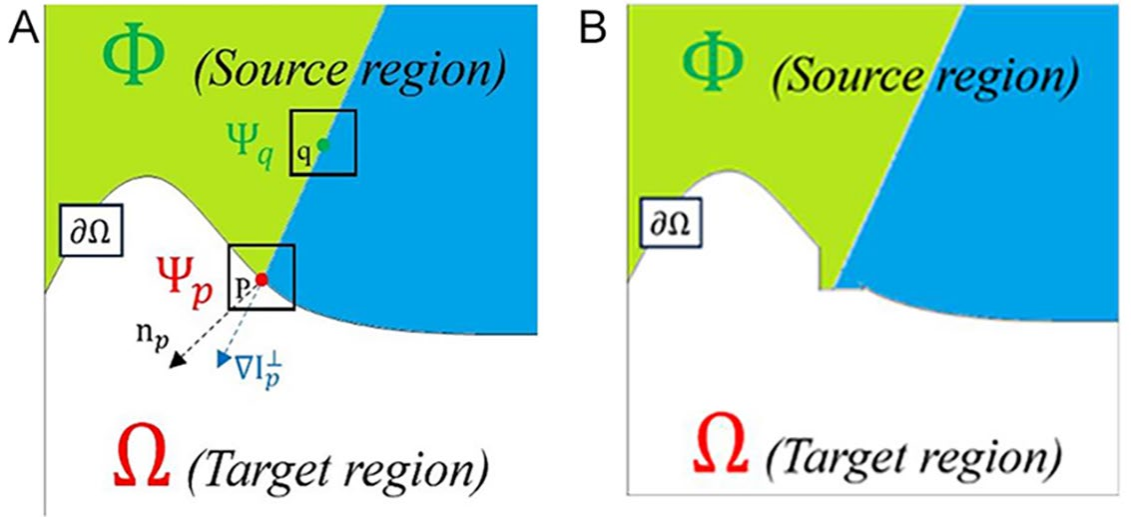
Schematic diagram of the Criminisi algorithm principle. **(A)** Before repair. **(B)** After repair.

After completing the software annotation, according to the dataset production process, the images need to be de-labeled to obtain images without black labels and corresponding json files. For image de-labeling, this paper makes improvements based on the Criminisi image restoration algorithm (Yang et al., 2020; Li et al., 2024; Yao, 2019). The Criminisi algorithm is a patch filling method that determines the repair order by comparing the effective information of boundary pixels. It finds the optimal filling patch within the entire image range through matching calculation rules to complete the filling of the pixel, and then performs a boundary and priority update cycle after completing one filling. Figure 5 shows the repair process of pixel P using the Criminisi algorithm.

In Figure 5A, the curve ∂Ω represents the boundary between the normal region and the region to be repaired, the area Φ above the curve is the normal range, different colors represent different textures in the area. The area Ω below the curve is the target area to be repaired, and P is the pixel point on the boundary ∂Ω . $\Psi_{p}$ is the pixel block to be repaired centered on P, and $n_{p}$ is the unit normal vector of the boundary of the area to be repaired. $\nabla I_{p}^{⊥}$ is the unit isophote gradient vector of P, $\Psi_{q}$ is the pixel block searched for and most similar to $\Psi_{p}$ , and *q* is the center of $\Psi_{q}$ . Figure 5B shows the effect of repairing point P.

As filling progresses, the average confidence level for the region will gradually decrease and eventually reach zero. Since the priority calculation is based on the product of the two values, this inevitably leads to the priority calculation failing to accurately reflect the regional information, resulting in calculation failure. This prevents the repair sequence from being reasonably arranged, ultimately leading to poorer repair results. To address this issue, the priority calculation method was improved by changing the priority calculation from a product form to an exponential sum form with base *e* (Jin et al., 2023), and limiting the confidence level to a certain range to avoid priority failure caused by low confidence levels. Equations (1)–(3) are the improved priority calculation Equations:


$$P(p)=e^{C(p)+D(P)}$$ (1)

$$C(p)=\{\begin{array}{c} \frac{\sum_{q\in \Psi_{p}\cap \Phi }C(q)}{|\Psi_{p}|}，C(p)>k_{\mathrm{thr}}\\ k_{\mathrm{thr}}，C(p)\leq k_{\mathrm{thr}} \end{array}$$ (2)

$$D(p)=\frac{|\nabla I_{p}^{⊥}\cdot n_{p}|}{\alpha }$$ (3)

When performing matching calculations, the Criminisi algorithm does not consider the texture characteristics of the areas to be repaired and the normal areas, and is somewhat blind. It only mechanically repeats the matching of texture features through matching rules, requiring traversing all pixels in the normal area of the image space, resulting in excessive computing costs. We adopt an improved matching mechanism based on the elliptic model, equivalent the marked region to the elliptic model, dividing the region into four regions along the elliptic symmetry axis, and limiting the entire matching search region within a reasonable range to seek the local optimal solution within this range (Fan et al., 2025). Therefore, when performing similarity matching calculations to find patches to fill in, prioritizing nearby areas for screening can not only effectively shorten the matching time, but also reduce the problem of pixel texture changes caused by mismatches.

This de-labeling algorithm is an improvement based on Criminisi. It calculates the average peak signal-to-noise ratio (PSNR) and structural similarity index measure (SSIM) between the images processed by the two algorithms to illustrate the differences in image restoration quality and conducts subjective evaluation through subjective visual effects. Equations (4) and (5) are respectively the calculation formulas for PSNR and SSIM:


$$\text{PSNR}=10\times [\log_{10}\frac{{\left(I_{r}^{\max}\right)}^{2}}{\mathrm{MSE}}+\log_{10}\frac{{\left(I_{g}^{\max}\right)}^{2}}{\mathrm{MSE}}+\log_{10}\frac{{\left(I_{b}^{\max}\right)}^{2}}{\mathrm{MSE}}]$$ (4)

$$\text{SSIM}(x,y)=l(x,y)\times c(x,y)\times s(x,y)=\frac{\left(2\mu_{x}\mu_{y}+c_{1})(2\sigma_{\mathrm{xy}}+c_{2}\right)}{\left(\begin{array}{l} \mu_{x}^{2}+\\ \mu_{y}^{2}+c_{1} \end{array})(\begin{array}{l} \sigma_{x}^{2}+\\ \sigma_{y}^{2}+c_{2} \end{array}\right)}$$ (5)

In these formulas, $I_{r}^{\max}$ , $I_{g}^{\max}$ , $I_{b}^{\max}$ represent the maximum pixel values for their respective channels, and $u_{x}$ , $u_{y}$ represents mean values for x and y respectively, $\sigma_{x}$ , $\sigma_{y}$ , denotes variance, and denotes covariance, $c_{1}={\left(k_{1}L\right)}^{2}$ , $c_{2}={\left(k_{2}L\right)}^{2}$ , $k_{1}$ and $k_{2}$ take values of 0.01 and 0.03 respectively, represents dynamic range of the images.

Since the number of acupoint markers is positively correlated with the image processing time, the processing speed of the algorithm for a single acupoint is defined as the average processing efficiency (APE) (unit: seconds per acupoint), as shown in Equation (6):


$$\mathrm{APE}=\frac{1}{n}\sum_{i=1}^{n}T_{i}$$ (6)

In the formula, $T_{i}$ represents the total time used by the algorithm to process a single image.

To evaluate the difference in processing efficiency between the Criminisi algorithm and the improved algorithm, we use the average efficiency improvement metric as defined in Equation (7).


$$\bar{\eta }=\frac{1}{n}\sum_{i=1}^{n}\eta_{i}=\frac{1}{n}\sum_{i=1}^{n}(1-\frac{T_{i}^{p}}{T_{i}^{c}})\times 100\%$$ (7)

In the formula, $T_{i}^{c}$ , $T_{i}^{p}$ represent the processing time of Criminisi and improved algorithm for single-image processing respectively, n is the total number of samples, and $\eta_{i}$ represents the efficiency improvement for a single image.

To enhance the diversity of the dataset and thereby improve the generalization ability and recognition robustness of the model, we adopted a single-graph data augmentation method, mainly achieved through random scale transformation, random clipping, and random horizontal flipping.

### 3.2 Acupoint recognition network model

The overall architecture of the network model in this study adopts a two-stage structure, that is, the combination of target range detection and key point detection for acupoint recognition. In this framework, the RTMDet network model (Bakhtiyorov et al., 2025; Lyu et al., 2022; Zhang et al., 2025) is first utilized to conduct object detection on the entire image and obtain the bounding box of the back area. Input this bounding box as ROI (region of interest) information into the RTMPose network model (Jiang et al., 2023; Kang et al., 2025; Zheng et al., 2025) to guide the subsequent key point prediction. The entire image still serves as the input for RTMPose, but the key point search range is effectively constrained by the bounding box, thereby reducing the interference of background noise and irrelevant information. The RTMDet model in the first stage is responsible for accurately locating the back region, enabling the prediction task in the second stage to focus on smaller and more relevant areas. The RTMPose model in the second stage conducts acupoint key point location on this basis, which can focus computing resources on key areas, thereby significantly improving prediction accuracy while ensuring reasoning speed.

**Figure 6:**
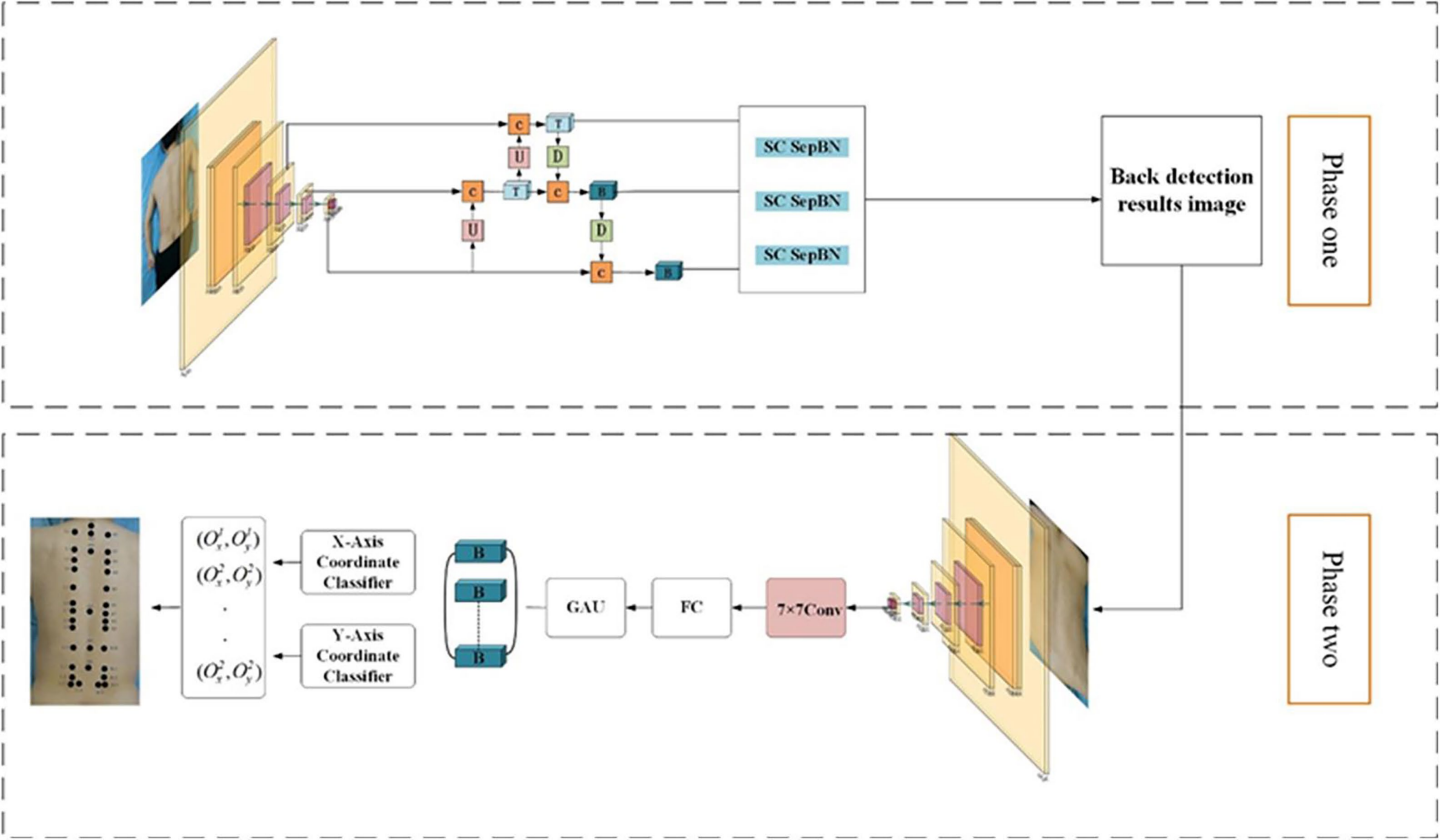
Two-stage acupoint recognition network structure.

The designed acupoint recognition network architecture is shown in Figure 6. Specifically, in the first stage, the RTMDet model is used to efficiently detect the patient’s back area. The CSPNeXt backbone network is used to extract multi-scale features, which are then combined with the PAFPN structure to enhance feature fusion capabilities. A dynamic label assignment strategy, SimOTA, is used to optimize sample matching, ultimately outputting precise back area bounding boxes and their corresponding positions in the camera coordinate system. The second stage is based on the RTMPose model to achieve accurate acupoint recognition. The GAU module (Hua et al., 2022) is the gate self-attention mechanism, which enhances the feature expression ability by combining the gating mechanism of GLU with single-head attention. Through the SimCC framework, coordinate prediction is converted into a sub-pixel classification task. Combined with the KL divergence loss function to optimize localization accuracy, the final result is the precise location of the acupoint. This solution maintains real-time performance while comprehensively balancing detection accuracy and computing efficiency.

This paper employs a learning rate scheduling strategy combining AdamW and Flat CosineLR. The first half adopts AdamW with a fixed learning rate of 0.05, and the second half adopts Flat CosineLR. AdamW replaces L2 regularization through weight decay, effectively preventing overfitting. Flat CosineLR first keeps the learning rate constant for a period of time (flat phase), then gradually reduces the learning rate to a minimum value. This strategy helps the model converge quickly in the early stages of training and fine-tune parameters in the later stages.

### 3.3 Evaluation indicator

Define the average pixel error for acupoint prediction in a single image $\mathrm{AP}E_{\text{acupoint}}$ , the actual error $\mathrm{APD}E_{\text{acupoint}}$ , the average recognition pixel error corresponding to the detection sample $\mathrm{mAP}E_{\text{acupoint}}$ , and the actual error $\text{mAPD}E_{\text{acupoint}}$ as evaluation indicators for acupoint recognition accuracy. The specific expressions are shown in Equations (8)–(13):


$$\mathrm{AP}E_{\text{acupoint}}=\frac{1}{m}\sum_{j=1}^{m}\max(P_{i,j,}-G_{i,j,2}-\frac{10}{k},0)$$ (8)

$$P_{i}=[\begin{array}{cc} x_{p}^{1} & y_{p}^{1}\\ x_{p}^{2} & y_{p}^{2}\\ \begin{array}{c} \vdots \\ x_{p}^{35} \end{array} & \begin{array}{c} \vdots \\ y_{p}^{35} \end{array} \end{array}]$$ (9)

$$G_{i}=[\begin{array}{cc} x_{g}^{1} & y_{g}^{1}\\ x_{g}^{2} & y_{g}^{2}\\ \begin{array}{c} \vdots \\ x_{g}^{35} \end{array} & \begin{array}{c} \vdots \\ y_{g}^{35} \end{array} \end{array}]$$ (10)

$$\mathrm{APD}E_{\text{acupoint}}=1.3\times \mathrm{AP}E_{\text{acupoint}}$$ (11)

$$\mathrm{mAP}E_{\text{acupoint}}=\frac{1}{n}\sum_{i=1}^{n}\mathrm{AP}E_{\text{acupoint}}^{i}$$ (12)

$$\text{mAPD}E_{\text{acupoint}}=\frac{1}{n}\sum_{i=1}^{n}\mathrm{APD}E_{\text{acupoint}}^{i}$$ (13)

Among them, $P_{i}$ and $G_{i}$ are, respectively, the predicted distribution matrix and the true distribution matrix of the acupoints in the i image, m is the number of identified acupoints, and n is the number of volunteers tested.

**Figure 7:**
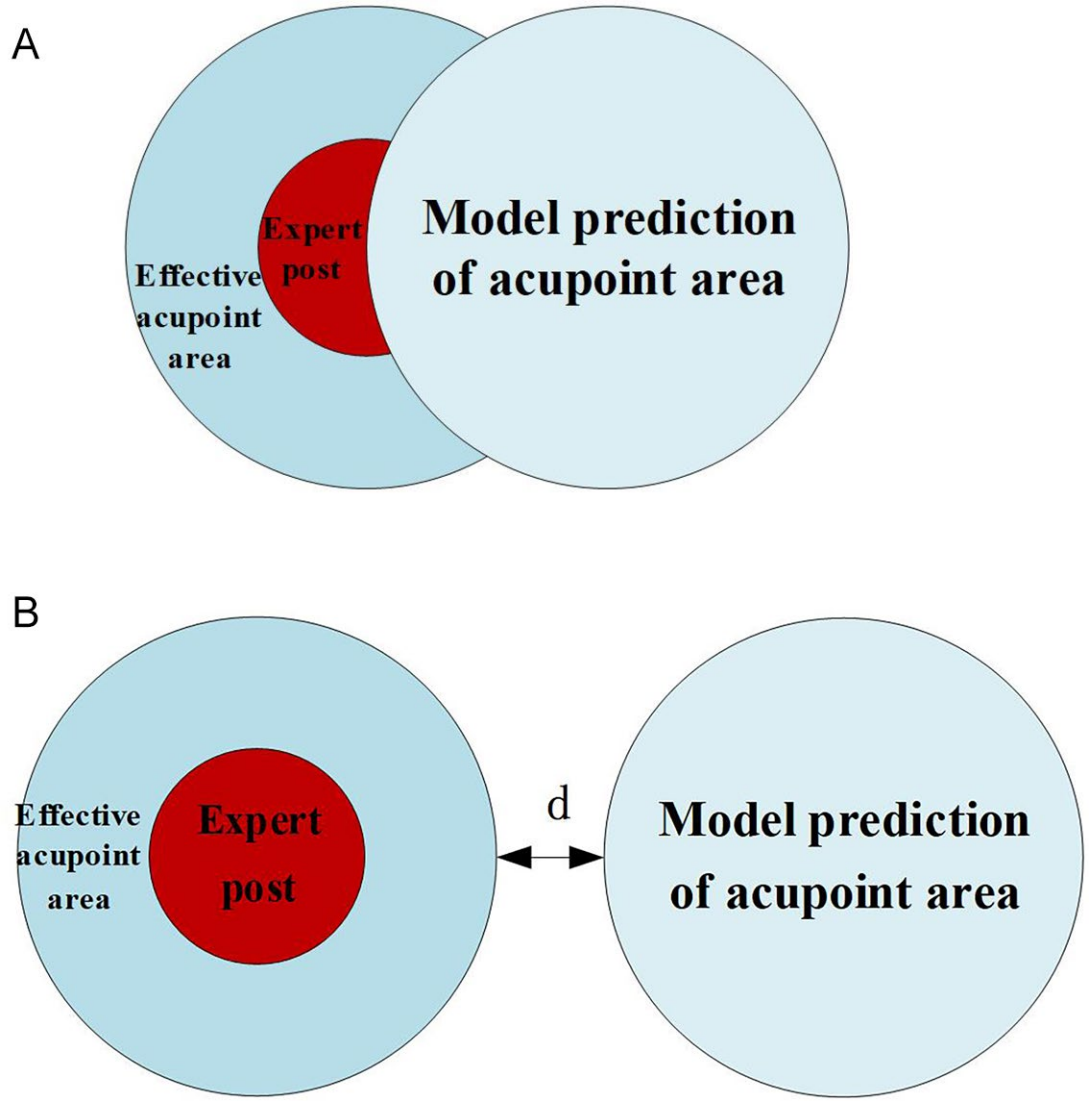
Relationship between model prediction and effective acupoint regions. **(A)** Effective identification (TP). **(B)** Existing error (FN).

Combining the acupoint patches used during data collection and acupoint selection, the area marked by experts is taken as the center of the effective acupoint area. The size of the effective acupoint region is set to a 10 mm circular area. The model predicts the acupoints to be the same 10 mm circular area. By analyzing the spatial relationship between the two circular regions, the error in the model’s predicted acupoints relative to the effective region is quantified. This defines the accuracy of acupoint recognition and the prediction error of acupoints, as shown in Figure 7.

If the predicted area of the model overlaps with the effective area of the label set, it is considered an effective recognition and is recorded as a positive example. If the two do not overlap, it indicates that there is an error in recognition and is recorded as a negative example. At this time, the error is denoted as *d*. When evaluating the model, recall is used to judge the overall accuracy of the model in acupoint recognition in a single image expressed by Equation (14).


$$\text{Recal}l_{\text{acupoint}}=\frac{1}{n}\sum_{i=1}^{n}\frac{\mathrm{TP}}{\mathrm{TP}+\mathrm{FN}}$$ (14)

where *n* is the number of volunteers tested, *TP* and *FN* correspond, respectively, to the positive and negative examples mentioned above, this formula indicates the average recognition accuracy of a single image.

### 3.4 Robot physiotherapy task planning

The coordinates of the acupoints output by the acupoint recognition model are the pixel coordinates of the acupoints in the image. During actual physiotherapy, the control of the physiotherapy end is based on the coordinates of the base coordinate system. Therefore, it is necessary to convert the recognized acupoints into the coordinates of the base coordinate system. Due to the different resolutions of depth cameras and color cameras, in order to obtain depth data at pixel coordinates, resolution conversion is required, that is, the registration of depth images and color images. The specific calculation method adopts the equivalence method, as shown in Equations (15) and (16). The image pixel coordinates are interpolated and filled through the normalization factor, that is, the color image coordinates correspond to the coordinates under the depth image, and the current depth value is the depth value z of the pixel coordinates under the color image.


$$u\prime =u\times s_{w},v\prime =v\times s_{h}$$ (15)

z(u,v)=d(u′,v′) (16)

Among them, $u,v,u\prime ,v\prime ,s_{w},s_{h},z,d$ represents the current pixel coordinates of the acupoint and the coordinates converted to the resolution of the depth map, the normalization factor, the current pixel coordinate depth value, and the depth data, respectively.

Then, convert the pixel coordinates to the camera coordinate system coordinates. Since the D435i camera is a planar camera, a central perspective model is adopted for modeling. The conversion relationship between image coordinates and camera coordinates is shown in Equations (17)–(20):


$$x_{c}=x\times \frac{z_{c}}{f}$$ (17)

$$y_{c}=y\times \frac{z_{c}}{f}$$ (18)

$$u=\frac{x}{\rho_{w}}+u_{0}$$ (19)

$$v=\frac{y}{\rho_{h}}+v_{0}$$ (20)

Thus, Equations (21) and (22) can be obtained.


$$x_{c}=\frac{(u-u_{0})\times \rho_{w}\times z_{c}}{f}$$ (21)

$$y_{c}=\frac{(v-v_{0})\times \rho_{h}\times z_{c}}{f}$$ (22)

The parameters such as focal length and principal point in the above calculation process can all be calibrated using the traditional checkerboard method based on Zhang Zhengyou’s calibration method, and can be obtained in the internal parameter matrix of the camera after calibration calculation,as shown in Equation (23).


$$K=[\begin{array}{ccc} \frac{f}{\rho_{w}} & 0 & 0\;\;u_{0}\\ 0 & \frac{f}{\rho_{h}} & 0\;v_{0}\\ 0 & 0 & \begin{array}{cc} 1 & 1 \end{array} \end{array}]$$ (23)

Among them, $f,\rho_{w},\rho_{h},(u_{0},v_{0})$ respectively represent the focal length of the camera, the width and height of each pixel point, and the coordinates of the principal point, which are the intersection points of the image plane and the optical axis.

Finally, after obtaining the coordinates in the camera coordinate system, they need to be converted to the coordinates in the base coordinate system and provided to the planning system for control, satisfying the following relationship as defined by Equation (24).


$$P_{\text{base}}=T_{c}^{b}P_{\text{camera}}$$ (24)

Among them, $P_{\text{base}}={\left(x_{b},y_{b},z_{b},1\right)}^{T}$ , $P_{\text{camera}}={\left(x_{c},y_{c},z_{c},1\right)}^{T}$ , $T_{c}^{b}$ are the homogeneous coordinate forms in the base coordinate system and the camera coordinate system respectively, as well as the homogeneous form of the coordinate transformation matrix between the camera coordinate system and the base.

**Figure 8:**
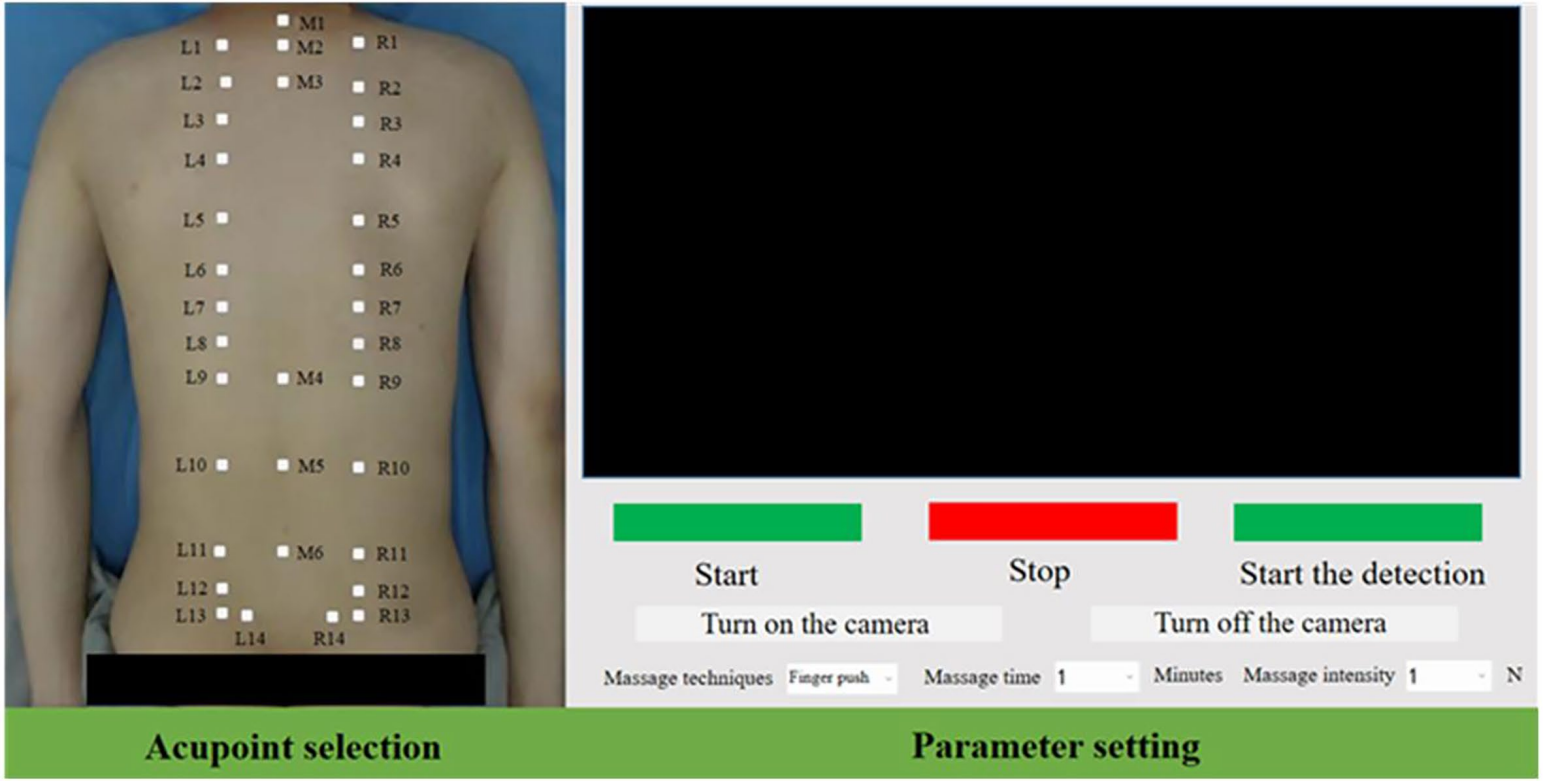
Control interface of acupoint recognition system.

According to the physiotherapy diagnosis plan, the operator sets the physiotherapy acupoints, physiotherapy techniques, as well as parameters such as force and time through the human-machine interface. As the actuator, the physiotherapy robotic arm reaches the acupoint recognition points in sequence for acupoint recognition. After the acupoint recognition is completed, it locates the acupoints to be physiotherapy one by one according to the names of the acupoints to be physiotherapy stored in the system and performs physiotherapy according to the set techniques. To facilitate the control of the physiotherapy process, the human-computer interaction interface design and control integration were carried out based on QT. The interface is shown in Figure 8.

After receiving the physiotherapy task, the entire process trajectory of the mechanical arm physiotherapy mainly consists of the following five steps,

1. The robotic arm moves from the initial posture to the posture for acupoint recognition.
2. The robotic arm moves from the position for acupoint recognition to above the first physiotherapy acupoint.
3. The robotic arm performs physiotherapy actions in accordance with the physiotherapy plan.
4. After the current acupoint physiotherapy is completed, proceed to the next physiotherapy acupoint.
5. After the current physiotherapy stage is completed, return to the physiotherapy point recognition point from the last acupoint.

To enhance the flexibility of human-computer interaction during physical therapy, different trajectory planning methods are adopted for trajectory control in each step. The first three steps and the fifth step are interpolated through the S-velocity curve planning algorithm in the joint Angle space to improve flexibility, while the fourth step uses the arc curve planning in Cartesian space.

## 4 Experiments and results

The recognition effect is mainly reflected in the accuracy and real-time performance of the key points recognition of physiotherapy. We integrated the acupoint recognition network into the physiotherapy robot system, and designed the accuracy and real-time related experiments of the key points recognition to verify the performance of the recognition system, and further carried out the physiotherapy task trajectory planning experiment. Verify the applicability and reliability of the entire detection system in the robot physiotherapy scenario.

Set up an acupoint recognition system test platform, install the D435i camera at the end of the robotic arm, set the height from the bed to 1 m, collect 40 data sets for acupoint accuracy testing and real-time detection efficiency, configure the computer with a 4070Ti GPU, set the camera resolution to 640 × 480, and measure that each pixel is converted to an actual distance of approximately *k* = 1.3 mm.

### 4.1 Result analysis and evaluation of the improved Criminisi algorithm

The marked images were processed one by one according to the Criminisi algorithm and the improved algorithm, and the experimental process data were recorded and saved. Calculate the PSNR and SSIM of the same image after processing by the two algorithms. The calculation results are shown in Table 1.

**Table 1:** Evaluation of algorithm repair efficiency.

Metrics name	$\bar{\text{SSIM}}$ (%)	$\bar{\text{PSNR}}$ (db)
Value	0.989946	37.646238

As can be seen from Table 1, the SSIM of the images processed by the two algorithms is relatively high, while the PSNR is close to 40db. The evaluation of these two objective indicators indicates that the restoration quality of the previous two algorithms is similar, but there are differences.

**Figure 9:**
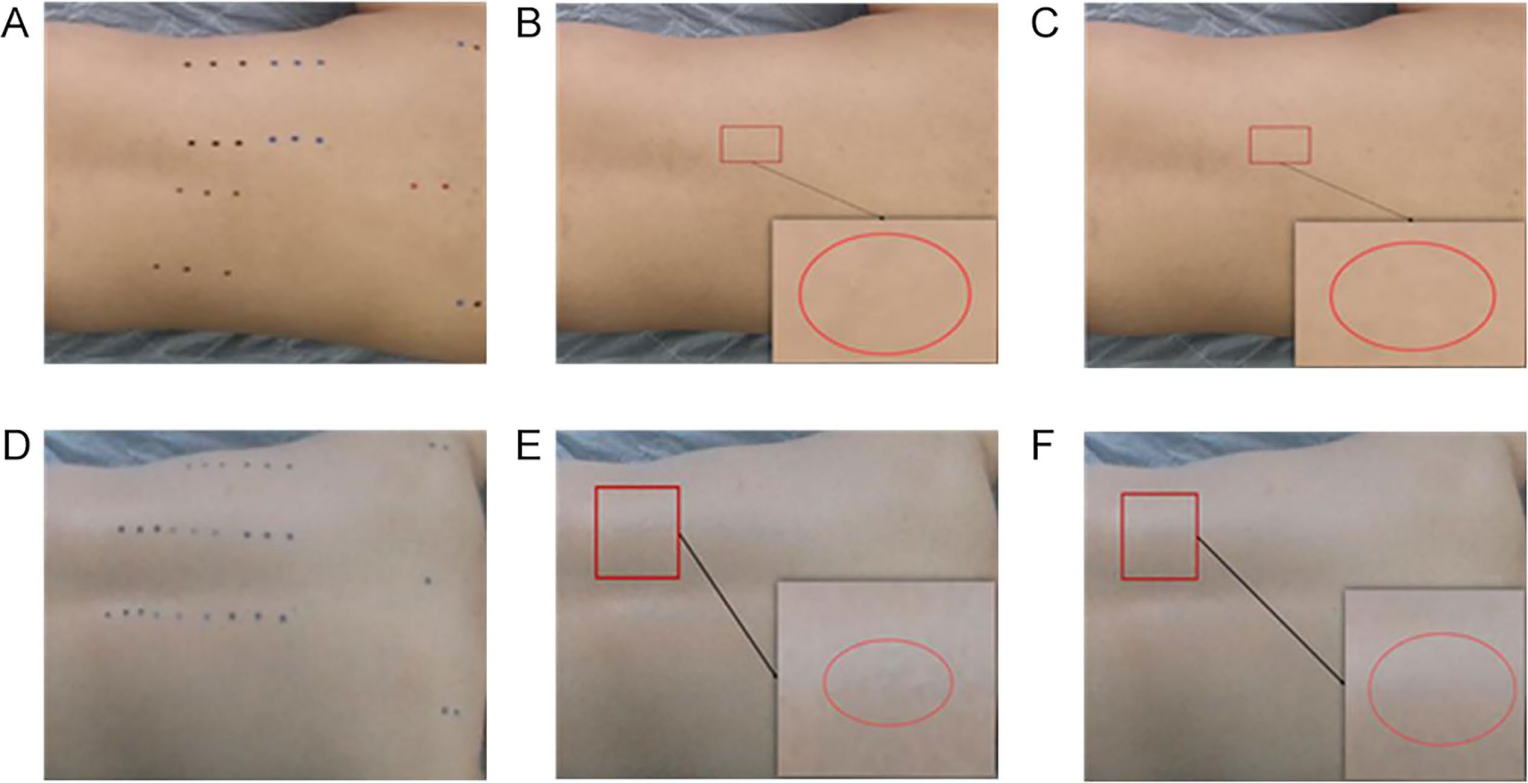
Comparison of the repair effects of the de-labeling algorithm. **(A)** Sample I. **(B)** Criminisi algorithm. **(C)** Improved algorithm. **(D)** Sample II. **(E)** Criminisi algorithm. **(F)** Improved algorithm.

In Figure 9, Figures 9B,C are the result graphs obtained by de-labeling Figure 9A with Criminisi algorithm and improved algorithm respectively, and Figures 9E,F are the result graphs obtained by de-labeling Figure 9D with Criminisi algorithm and improved algorithm respectively. By comparing the images before and after processing and their local magnifications, it can be found that the Criminisi algorithm is prone to problems such as discontinuous boundaries and pixel mutations between different patches after restoration. Under the same conditions, the improved algorithm effectively alleviates the above phenomenon, making the repaired area and the surrounding skin appear more natural and the transition smoother subjectively visually. Since the application scenario of this study is acupoint recognition, there are high requirements for the coherence and naturalness of the repair area. Therefore, the proposed algorithm performs more superior in meeting this demand.

**Figure 10:**
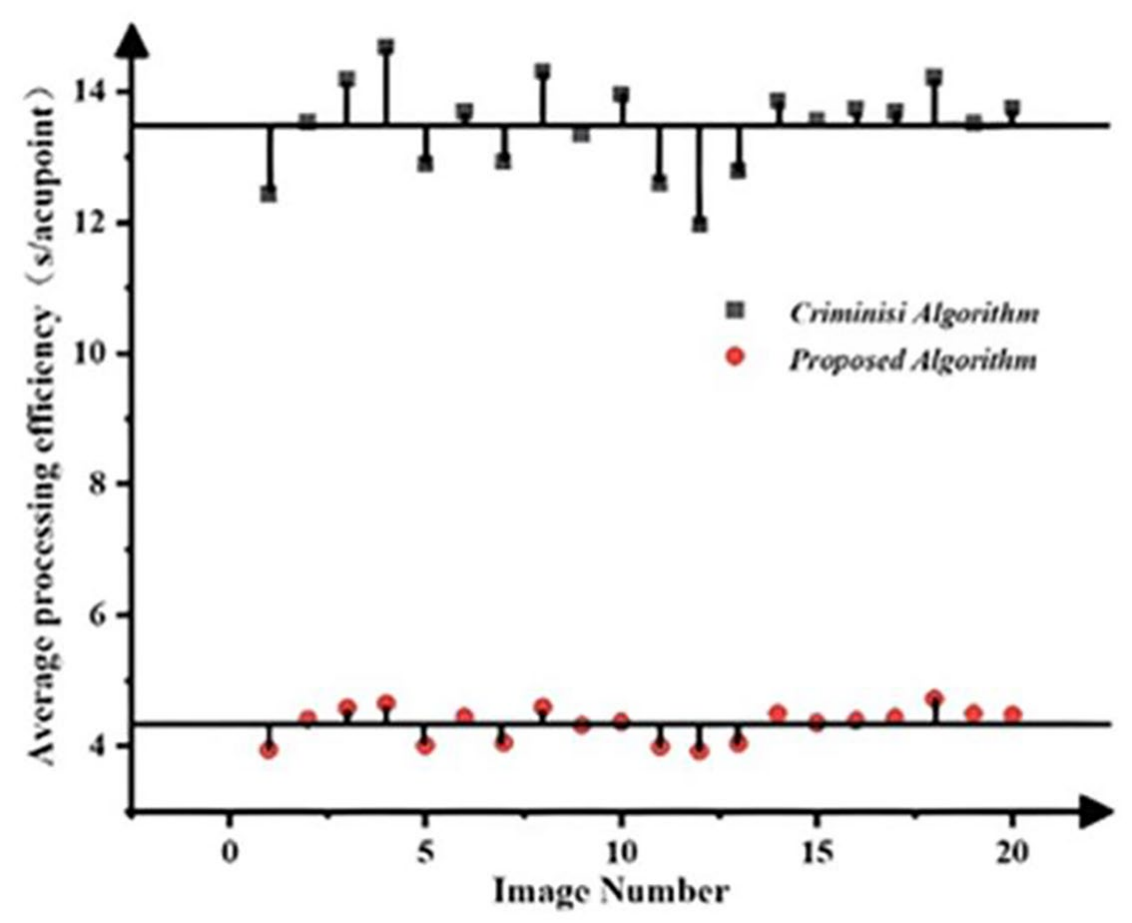
Comparison of algorithm repair efficiency.

Calculating the APE and $\bar{\eta }$ for the Criminisi and improved algorithms, and plot the APE of the algorithms as shown in Figure 10, the algorithm repair efficiency evaluation table is shown in Table 2.

**Table 2:** Evaluation of algorithm repair efficiency.

Algorithm name	APE (s/acupoint)	$\bar{\eta }$ (%)
Criminisi algorithm	13.4817	/
Proposed algorithm	4.333	67.8

Firstly, as can be seen from Figure 10, the APE of the Criminisi algorithm is significantly greater than that of the improved algorithm, indicating that the improvement of the matching mechanism by the algorithm can effectively enhance the computational efficiency of the algorithm and save processing time. Moreover, the processing efficiency of each single sheet of the algorithm fluctuates around a certain mean value, and the fluctuations are relatively small, indicating strong stability of the algorithm. Secondly, as shown in Table 2, the average APE of the Criminisi algorithm is 13.4817. After the improvement, the average APE of the algorithm is reduced by 9.1487 compared with the former, and the algorithm efficiency is increased by 67.8%. Therefore, the improved algorithm can effectively increase the speed of acupoint de-labeling processing and has strong practical significance for the processing of large-scale acupoint datasets.

### 4.2 Accuracy test for acupoint recognition

To evaluate the accuracy of the acupoint recognition method, this study selected several volunteers as test subjects, collected predicted coordinates, and calculated evaluation indicators.

**Figure 11:**
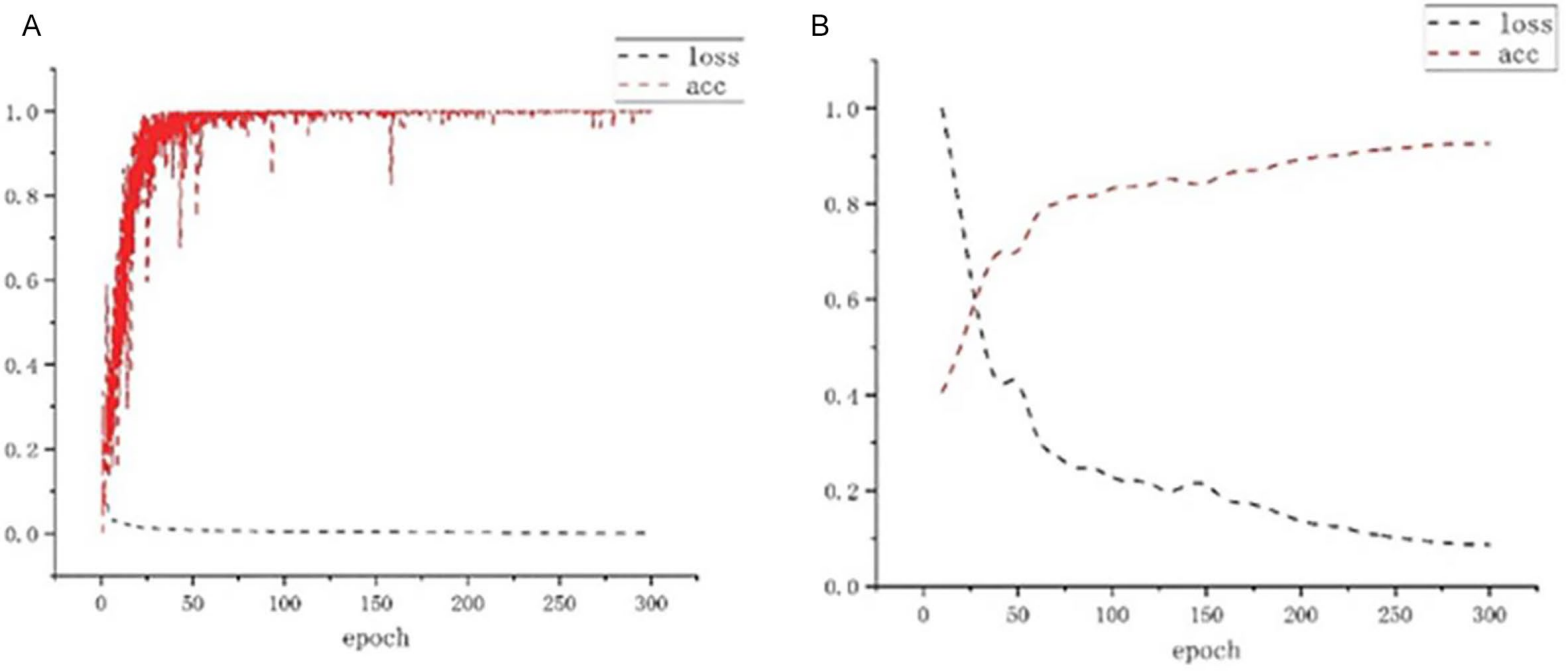
Loss and acc change diagram. **(A)** Training set loss and accuracy. **(B)** Validation set loss and accuracy.

**Figure 12:**
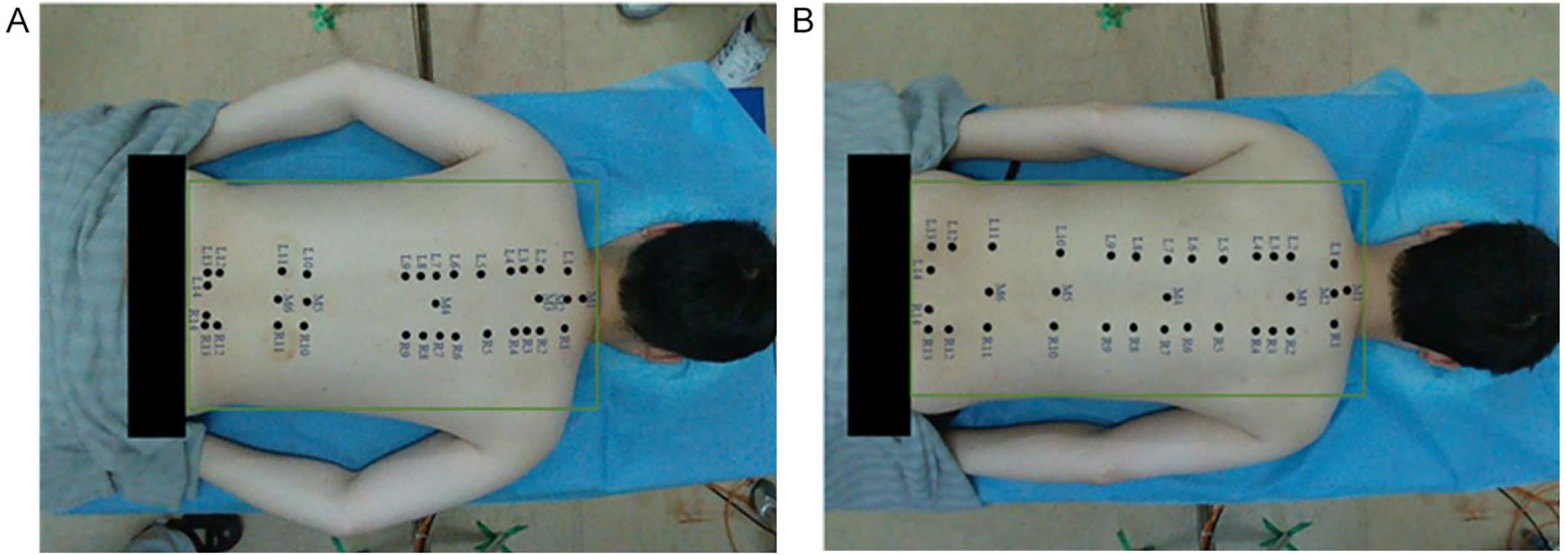
Acupoint recognition effect diagram. **(A)** Example diagram Ⅰ. **(B)** Example diagram Ⅱ.

A total of 1,000 high-quality datasets were selected from the dataset and distributed in a ratio of 8:2 between the training set and the validation set. Each was trained for 300 rounds, and the validation set error was calculated every 10 rounds. The training results of the acupoint detection model are shown in Figure 11. As can be seen from Figure 11A, the accuracy is already relatively high around 50 rounds. After 300 rounds of training, the model’s accuracy in the training set is close to 1. From Figure 11B, the accuracy in the validation set reaches over 80% around 150 rounds, which is relatively high. After 300 rounds of training, the accuracy reaches over 90%. Figure 12 shows the prediction results of the acupoint recognition model after training. M1-M6 are the acupoints in the middle of the human back, L1–L14 are the acupoints on the left side of the human back, and R1–R14 are the acupoints on the right side of the human back.

We conducted an average statistical analysis based on 40 samples. To demonstrate the representativeness of the results, the relevant parameters of five samples and the average statistical results of the overall sample are listed in Table 3. Through the evaluation of the positioning accuracy of the model, the average pixel error of acupoint recognition is 4.45 pixels. After the scale coefficient *k* conversion, the corresponding actual physical error is approximately 5.78 mm, the sample variance is 2.91 and the average recall rate reaches approximately 90.17%. During the execution of the task, this error mainly stems from the positioning error of the X-axis and Y-axis and the approximate error of proportional geometric measurement. Meanwhile, in the Z-axis, it also includes the error of sensor depth information. The results show that this detection method has high accuracy and can meet the application requirements of most physiotherapy scenarios such as massage and moxibustion.

**Table 3:** Performance of the sample test set for acupoint accuracy indicators.

Sample	1	2	3	4	5
$\mathrm{AP}E_{\text{acupoint}}^{i}$ (pix)	4.14	2.13	2.76	5.21	4.91
$\mathrm{APD}E_{\text{acupoint}}^{i}$ (mm)	5.34	2.74	3.56	6.72	6.33
$\mathrm{mAP}E_{\text{acupoint}}$ (pix)			4.45		
$\text{mAPD}E_{\text{acupoint}}$ (mm)			5.78		
$\text{Recal}l_{\text{acupoint}}$ (%)			90.17		

We also measured the average error data and related analysis contents of the relevant acupoints of 20 subjects, and calculated the accuracy of each acupoint one by one. The accuracy statistics of the acupoints located on the spine are shown in Table 4, and the accuracy statistics of the acupoints on both sides of the spine are shown in Table 5. Here, the acupoints at the same height on both sides of the spine were taken as the acupoint pairs for average calculation. Among them, the accuracy rate of acupoints located on the spine is generally higher than that of acupoints on both sides of the spine. Meanwhile, the accuracy rate of M1 and M2 acupoints is higher than that of M5 and M6 acupoints. This is because during the process of experts marking acupoints, they often take M1 and M2 acupoints as the marking benchmarks and give priority to marking them. From the perspective of human musculoskeletal system, the characteristics of these two acupoints are more obvious compared to M5 and M6 acupoints. For acupoints with weaker characteristics, the detection difficulty is greater. Meanwhile, experts often mark the acupoints on the spine first and then look for the pairs of acupoints on both sides, especially for those of the same height. The accuracy of the pairs of acupoints on both sides is directly related to the accurate values of the acupoints on the spine.

**Table 4:** Accuracy test statistics of acupoints in the middle of the spine.

Acupuncture point	M1	M2	M3	M4	M5	M6
$\text{mAPD}E_{\text{acupoint}}$ (mm)	2.34	2.45	3.66	4.43	4.94	5.96

**Table 5:** Accuracy test statistics of acupoints on both sides of the spine.

Acupuncture point	L1 & R1	L2 & R2	L3 & R4	L4 & R4	L5 & R5	L6 & R6	L7 & R7	L8 & R8	L9 & R9	L10 & R10	L11 & R11	L12 & R12	L13 & R13	L14 & R14
$\text{mAPD}E_{\text{acupoint}}$ (mm)	2.88	3.09	3.78	5.62	6.84	5.39	4.93	5.67	5.79	5.79	6.01	5.82	5.32	4.59

### 4.3 Real-time testing of acupoint recognition

In physical therapy scenarios, due to the movement of the patient’s body and changes in lighting, it is necessary to constantly update the location of acupoint recognition. Therefore, the acupoint recognition system must have the ability to update detection in real time. To evaluate this real-time performance, the detection speed of the acupoint recognition, that is, the number of image frames inferred per second, is used as the real-time evaluation index for acupoint prediction. Volunteers were arranged to dynamically move their body postures, and the detection effects of different body postures were tested.

**Figure 13:**
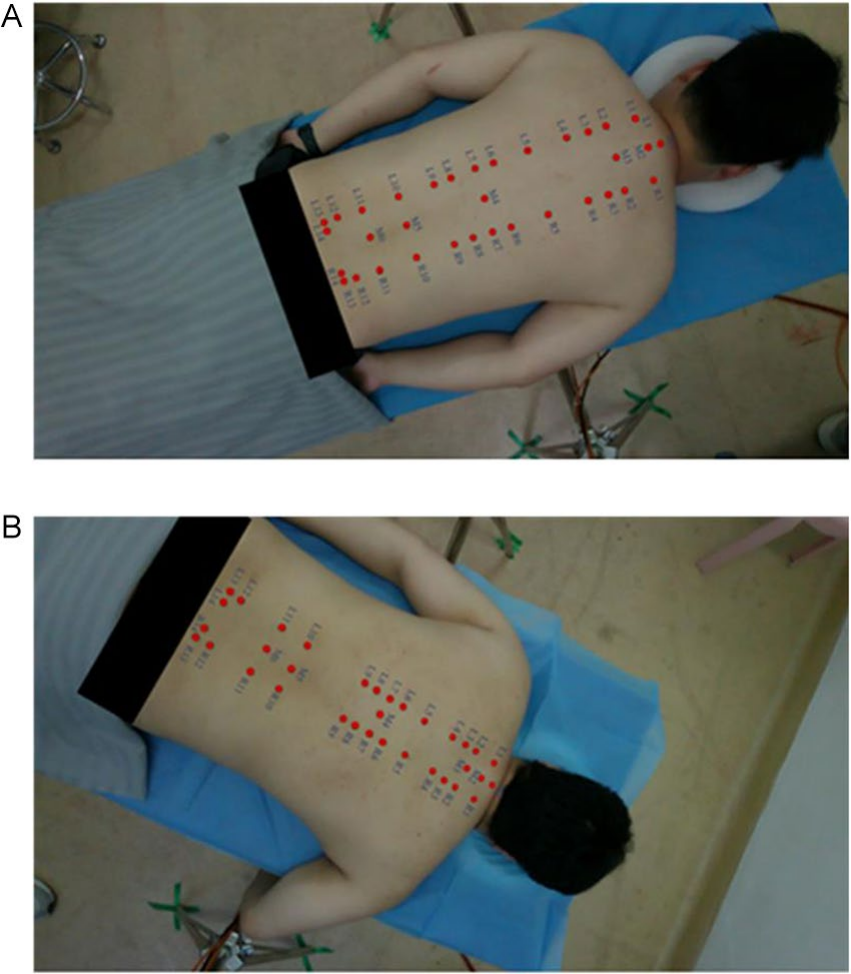
Recognition effect diagram. **(A)** Example diagram Ⅰ. **(B)** Example diagram Ⅱ.

The detection results of the acupoint recognition model are shown in Figure 13, where the red areas indicate the acupoint locations predicted by the model. From left to right are dynamic acupoint recognition images of the back under different postures. After stabilization, the number of detection frames was recorded, and the average value was calculated to be 30 (FPS). It can be seen that the detection results have a very high detection efficiency, and this method has good applicability.

### 4.4 Experiment on trajectory planning for physiotherapy tasks

**Figure 14:**
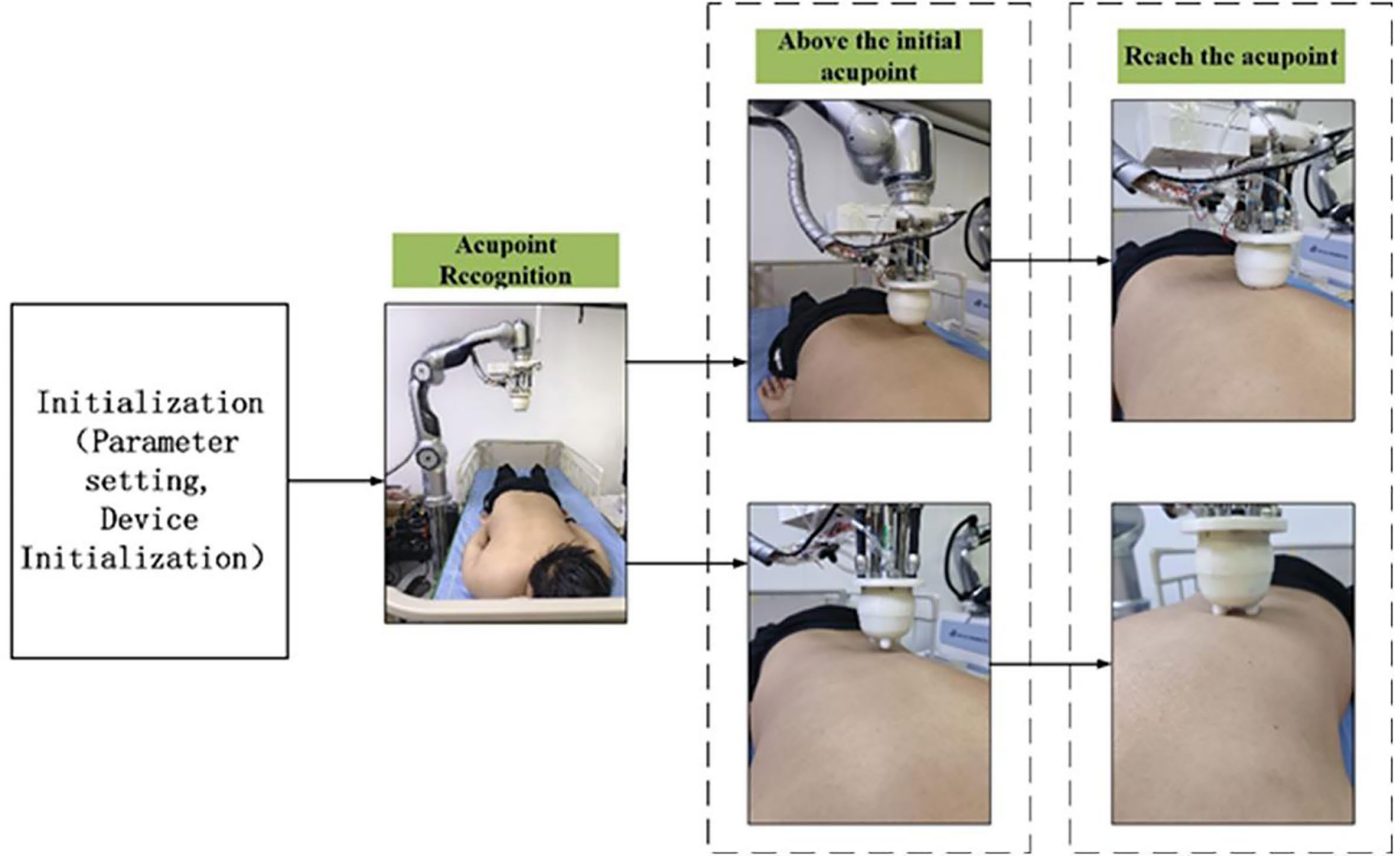
Physiotherapy process.

Through the human-machine interface operation, parameters such as the physiotherapy technique, duration and intensity were selected, and then the physiotherapy began. The experimental process is shown in Figure 14.

**Figure 15:**
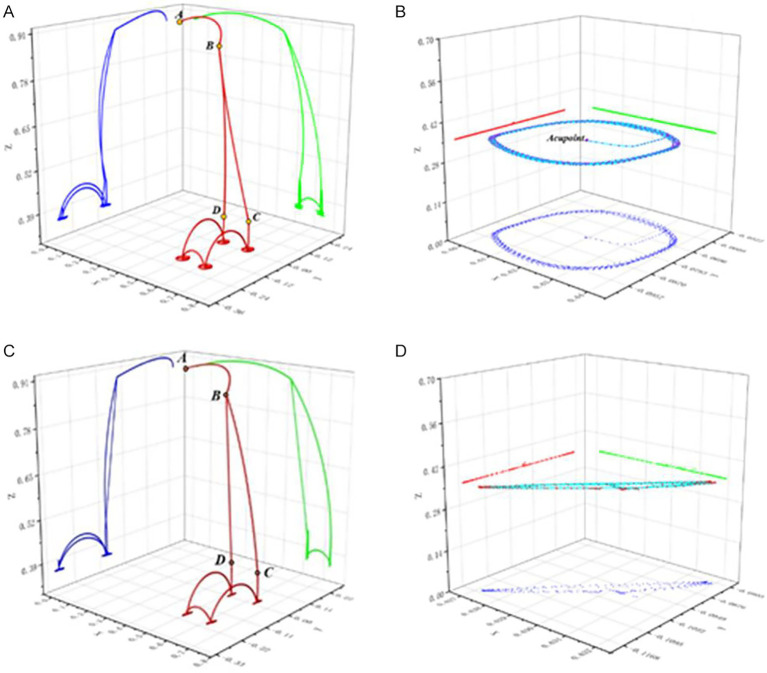
Physiotherapy task planning trajectory diagram. **(A)** The entire process trajectory of physiotherapy task planning I. **(B)** The typical trajectory of physiotherapy task planning I. **(C)** The entire process trajectory of physiotherapy task planning II. **(D)** The typical trajectory of physiotherapy task planning II.

In this section, four acupoints on the back were selected for the full-process physiotherapy trajectory planning experiment. Four techniques were planned in sequence, and the end poses and trajectory planning data were recorded. The full-process trajectories of each technique and the typical trajectories of the techniques are shown in Figure 15. Point A is the system initialization pose location, and point B is the waiting point for acupoint recognition. The system moves from point A to point B for acupoint recognition. After the recognition is completed, points C and D are selected based on the chosen acupoints, which are above the acupoints to be treated. These points are, respectively, the first and last acupoints to be treated. The AB, BC, and CD sections are planned using the S-speed curve.

### 4.5 Comprehensive analysis

In Table 6, we made a comprehensive comparison between the proposed method and the existing acupoint recognition methods from four dimensions of positioning accuracy, convenience of use, cost, scope of application. From the comparison results, it can be seen that although the method in literature (Zheng, 2022) stands out in terms of convenience, cost and has a wide scope of application, its positioning accuracy is relatively low. The method in literature (Wang et al., 2023) has high positioning accuracy but has the shortcomings of inconvenience in use, high cost and limited scope of application. The method in literature (Zhang et al., 2024) has advantages in convenience and cost. However, the positioning accuracy is moderate and the scope of application is limited. Overall, our method achieves a more balanced performance in these four dimensions, with more competitive comprehensive performance, providing a more practical solution for acupoint recognition.

**Table 6:** Comprehensive performance comparison table of acupoint recognition methods.

Evaluation criteria	Zheng (2022)	Wang et al. (2023)	Zhang et al. (2024)	Our
Positioning accuracy	Low	High	Medium	High
Convenience	High	Low	High	High
Cost	Low	High	Low	Low
Scope of application	Widely	Limit	Limit	Widely

We placed the proposed method and the current mainstream key point detection methods under the same experimental conditions for performance verification, using exactly the same hardware configuration and strictly following the same data preprocessing process to eliminate the interference of experimental environment differences on the evaluation results. The comparative experiment adopts the test dataset. The acupoint recognition accuracy of the proposed method is quantified by calculating the acupoint prediction error (APE) corresponding to all effective pixels, and the detection speed of acupoint recognition, that is, the number of image frames per second (FPS), is calculated to quantify the real-time performance of acupoint recognition of the proposed method. It can be seen from Table 7 that the method we proposed performs best on APE, significantly lower than other methods. In terms of reasoning efficiency, although it is slightly lower than YoloV8, it is higher than the method based on human body model, and the gap with RTM-pose is also within an acceptable range. On the whole, our method has achieved a good balance between accuracy and efficiency, and has an advantage in the accuracy of acupoint coordinate prediction. It can better meet the high requirements for acupoint recognition accuracy in physiotherapy scenarios and lay a solid foundation for the precise execution of subsequent physiotherapy actions.

**Table 7:** Comparison table of acupoint recognition accuracy and speed.

Method	APE	FPS
YoloV8	8.94	59
Method based on human body model	10.89	10
RTM-pose	6.52	36
Our	5.78	30

## 5 Discussion

This paper conducts research on the autonomous acupoint recognition of physiotherapy robots. In response to the difficulties such as the dense distribution of acupoints on the back, the indistinct external features, and the changes in posture and lighting, a two-stage detection method combining RTMDet and RTMPose is proposed, and model generalization is carried out for acupoint recognition under dynamic conditions. Through experimental verification, we demonstrate the robustness and practicality of the proposed method in complex environments. In the task of locating acupoints, accurately identifying the positions of key points depends not only on local texture features, but also on the overall structure and contextual semantic information. Traditional single-stage networks often struggle to strike a good balance between ensuring target detection and precise prediction of key points simultaneously. The two-stage architecture effectively alleviates this contradiction by decoupling target detection from key point localization. The first stage of the RTMDet network model focuses on the back region of the human body, eliminating redundant interference information; while the second stage of the keypoint localization network RTMPose focuses on high-precision acupoint recognition tasks within the cropped local region. Through module division and task decoupling, this structure not only improves the robustness and generalization ability of the model, but also provides better spatial constraints and feature support for precise acupoint recognition in complex contexts. Based on the defined accuracy error metric, it achieves a recall of 90.17% on the human body dataset, with a detection error of around 5.78 mm. Moreover, it can still conduct real-time detection when the volunteer moves their body and their posture changes, and the accuracy is almost unaffected. The average frame rate remains above 30 frames, indicating that the system can meet the requirements of real-time application scenarios. However, it should be noted that since the data collection is carried out in a well-lit and controllable laboratory environment, which differs from the actual application scenarios, different light intensities may have a certain impact on the accuracy of acupoint recognition. Moreover, the data of this study mainly came from subjects with healthy body types and did not include data cases of diseases such as “scoliosis.” Therefore, when it comes to users with similar physical disabilities and scoliosis, the generalization is reduced. In future research, we plan to introduce other perception methods to build a multimodal fusion acupoint recognition system, in order to enhance the adaptability and accuracy of the model in complex clinical scenarios. Meanwhile, we have expanded our research scope from the back to other parts of the human body such as the abdomen and ears, gradually establishing a high-quality database of acupoints throughout the body.

## 6 Conclusion

In response to the imbalance between the demand and supply of physical therapy for the aging and sub-healthy population, the application of robots for physical therapy has become a highly promising solution. However, the current level of automation in acupoint recognition of physical therapy robots is insufficient, and there is still room for improvement in recognition accuracy. In terms of dataset collection and production, a high-precision dataset production method was designed, which involves manual labeling first and then marking through algorithms, thereby improving the quality of the dataset. Based on the deep learning algorithm model, a two-stage acupoint recognition model is proposed. Object detection adopts network architectures such as CSPNeXt and enhances detection speed and accuracy through a multi-head sharing mechanism. Key point detection is based on the SimCC architecture, converting regression problems into classification problems, which improves detection speed and raises detection accuracy to the sub-pixel level. By transforming the coordinates, we planned the task execution trajectory of the physiotherapy robot to ensure that it could precisely reach the target acupoints when performing physiotherapy actions. The algorithm model has been verified. The average pixel error of acupoint recognition is 4.45 pixels, the actual physical error is about 5.78 mm, and the average recall rate is about 90.17%. This indicates that the detection model method has a high detection accuracy and can meet the needs of most physiotherapy scenarios. This research is conducive to promoting the deep integration of traditional Chinese medicine and artificial intelligence technology, and driving the development of physiotherapy robots.

## Data Availability

The raw data supporting the conclusions of this article will be made available by the authors, without undue reservation.
